# Affecting Factors of Plant Phyllosphere Microbial Community and Their Responses to Climatic Warming—A Review

**DOI:** 10.3390/plants12162891

**Published:** 2023-08-08

**Authors:** Shaolin Huang, Xinjie Zha, Gang Fu

**Affiliations:** 1 Lhasa Plateau Ecosystem Research Station, Key Laboratory of Ecosystem Network Observation and Modeling, Institute of Geographic Sciences and Natural Resources Research, Chinese Academy of Sciences, Beijing 100101, China; huangsl.20b@igsnrr.ac.cn; 2 Xi’an University of Finance and Economics, Xi’an 710100, China; zhaxj@xaufe.edu.cn

**Keywords:** biodiversity, climate change, increased temperature, phyllosphere microbes, epiphytic microbes, endophytic microbes

## Abstract

Phyllosphere microorganisms are not only an important part of plants, but also an important part of microorganisms. In this review, the function of phyllosphere microorganisms, the assembly mechanism of phyllosphere microorganisms, the driving factors of phyllosphere microbial community structure, and the effects of climate warming on phyllosphere microbial community structure were reviewed. Generally, phyllosphere microorganisms have a variety of functions (e.g., fixing nitrogen, promoting plant growth). Although selection and dispersal processes together regulate the assembly of phyllospheric microbial communities, which one of the ecological processes is dominant and how external disturbances alter the relative contributions of each ecological process remains controversial. Abiotic factors (e.g., climatic conditions, geographical location and physical and chemical properties of soil) and biological factors (e.g., phyllosphere morphological structure, physiological and biochemical characteristics, and plant species and varieties) can affect phyllosphere microbial community structure. However, the predominant factors affecting phyllosphere microbial community structure are controversial. Moreover, how climate warming affects the phyllosphere microbial community structure and its driving mechanism have not been fully resolved, and further relevant studies are needed.

## 1. Introduction

**Figure 1:**
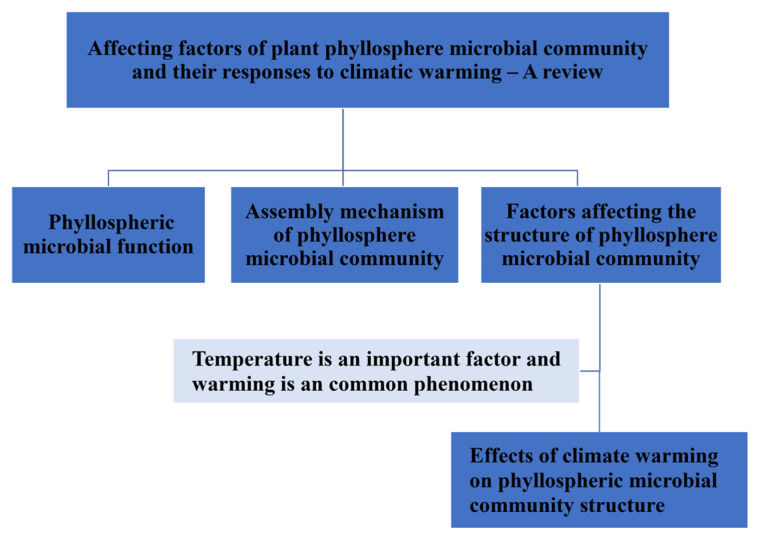
SmallArt for the relationships among the main contents in this study.

With the development of microbiome-related technology and human understanding of the multiple functions of phyllosphere microorganisms, a great deal of studies have been carried out around phyllosphere microorganisms. In order to reach more general conclusions and provide better services for food production and foliar microbial scientific research, this study conducted a systematic review on four aspects (Figure 1). First, we introduced the multiple functions of phyllospheric microorganisms. Second, the community assembly mechanism of phyllosphere microorganisms was reviewed. Third, the influencing factors of phyllosphere microorganisms were reviewed. Fourthly, the effects of climate warming on phyllosphere microorganisms were reviewed.

There are still some questions which need to be further studied, and future scientific research can be carried out on the following aspects. First, what are the main ecological processes involved in the assembly of a phyllosphere microbial community, and moreover, do external disturbances alter the relative effects of various ecological processes on the assembly of phyllospheric microbial communities? Second, what are the dominant factors in the community structure of phyllosphere microbiota? Third, how will climate warming change phyllosphere microbial communities?

## 2. Phyllospheric Microbial Function

Phyllosphere microorganisms have a variety of functions, such as degrading environmental pollutants, fixing nitrogen, promoting plant growth, inhibiting pathogenic microorganisms and improving plant resistance to pathogens, etc. Some components of phyllosphere microorganisms can cause plant diseases, and even lead to poisoning, allergy and infection reactions in humans and animals [1,2]. Using the beneficial functions of the phyllosphere microorganisms to promote plant growth is a sustainable solution [2,3,4,5,6,7]. In agricultural ecosystems, the biological potential of beneficial bacteria (biocontrol bacteria, plant growth hormone producing bacteria and azotobacter, etc.) in phyllosphere microorganisms should be fully tapped. For example, biocontrol bacteria isolated from the plant phyllosphere can be used to prevent and control plant phyllosphere diseases. Moreover, phyllosphere functional traits can be regulated by phyllosphere microorganisms to improve plant adaptability. This will help reduce fertilizer and pesticide input, promote plant growth and reduce environmental pollution, and thus achieve high-quality agricultural development [2,3,4,5,6,7]. As important phyllosphere microorganisms, *Methylobacterium* spp., *Sphingomonas* spp., *Pseudomonas* spp., etc., are all potential beneficial bacteria, whereas *Alternaria* spp., *Fusarium* spp., etc., are potential pathogens.

*Methylobacterium* was proposed in 1976 and consists of Gram-negative bacteria belonging to the phylum alpha-Proteobacteria [8]. *Methylobacterium* is present in the phyllosphere of most plants and is dominant in the phyllosphere of rice, corn, soybean and clover [8,9,10]. They are able to use organic single-carbon compounds (such as formates, formaldehyde, methanol and methylamine, etc.) as the sole source of carbon and energy for their growth [8]. *Methylobacterium phyllophyllum* can regulate plant hormone levels by producing metabolites such as vitamin B12, plant growth hormone and cytokinin, and protect plants from pathogens, thus promoting plant growth and development [8,11,12]. Because of its polyketosynthase, *Methylobacterium phyllophyllum* can produce compounds that absorb ultraviolet light, so it can effectively resist harmful ultraviolet radiation [8,13]. Some plants, such as *M. populi*, *M. komagatae* and *M. aquaticum*, have acetylene-reducing activity, suggesting that they may have nitrogenase activity and may also be involved in nitrogen metabolism in plants through urease [14]. The content of protein related to methanol assimilation was higher in methyl interphyllum [10]. Therefore, *Methylobacterium interphylloides* can also degrade methanol on the surface of plant leaves (a byproduct of pectin demethylation during cell wall metabolism) [8,10] so as to alleviate the toxic effect of methanol on plants.

*Sphingomonas* exists in the phyllosphere of rice, corn, green onion, citrus and poplar [10,15,16]. *Sphingosine* with bacteria show resistance to environmental stresses of regulatory factors, so they can enhance the stress tolerance of plants [17]. *Sphingosinomonas* biodegrades xylene and 2,4-dichlorophenoxyacetic acid by releasing monooxygenase and dioxygenase to improve the drought resistance of plants. *Sphingomonas phyllosphaerae* can improve the ability of plants to resist phyllosphere pathogens by inhibiting the growth of pathogenic bacteria [18]. *Sphingomonas paucimobilis* can degrade organic pollution, thereby reducing the impact of pollutants on phyllosphere growth [19]. In addition, *Sphingomonas* has transporter-related proteins that transport sugars, vitamins and iron carriers, characteristics that may make *Sphingomonas* competitive with other phyllosphere microorganisms [10].

*Pseudomonas* is a kind of widely distributed Gram-negative bacteria, which can produce a variety of active metabolites, induce disease resistance in plants and thus resist the infection of pathogenic microorganisms. About half of the species in this genus produce surfactants that increase foliar wetting and thus alter the interfoliar water condition [20]. *Pseudomonas* also has the ability to protect against ultraviolet radiation [16].

*Streptospora* and *Fusarium* are common colonizing bacteria in the wheat phyllosphere, both of which can cause disease and produce mycotoxins that are harmful to consumers [21]. The wetter and colder the microclimate, the higher the *fusarium* spores and genetic abundance [21]. *Streptospora* showed the opposite trend in genetic abundance, while its spore deposition was not correlated with any microclimatic conditions and was more evenly distributed in the field [21].

*Rhizobium* spp. can promote plant growth by fixing atmospheric nitrogen or regulating the secretion of plant hormones [16,22]. *Rhizobium* spp. can also compete with leaf surface pathogens or regulate the onset and spread of disease by producing antibiotics or inducing plant immune responses [16,22]. *Azospirillum* spp. can use the nutrients and energy produced by the phyllosphere to play a nitrogen fixation role [23].

## 3. Assembly Mechanism of Phyllosphere Microbial Community

The mechanism of microbial community assembly has always been a hot scientific issue in the field of microbial ecology. Given the important agricultural and ecological significance of plant phyllosphere microorganisms, it is important to elucidate the relative importance of different ecological processes (such as selection and dispersal) involved in the assembly of plant phyllosphere microbial communities [24,25]. Moreover, it is also important to clarify how global changes affect the various ecological processes involved in the assembly of phyllosphere microbial communities [24,25].

At present, it is generally believed that microbial community assembly is regulated by both deterministic and stochastic processes [26,27]. The stochastic process is based on neutral theory, while the deterministic process is based on niche theory [28,29]. Niche theory holds that biological interactions (such as competition) and environmental filtering drive microbial community assembly [26,28,29]. However, neutral theory holds that all species or individuals in a community are ecologically equivalent, and that community structure is independent of species traits and governed by random processes of birth, death, colonization, extinction and speciation [26,28,29]. The mechanisms that form microbial species diversity are generally considered to be ecological processes, including selection, dispersal, diversification and drift. The selection process is often regarded as the determination process, whereas the processes of dispersal, diversification and ecological drift are regarded as random processes [26,30]. The selection process in the assembly of the phyllosphere microbial community includes host filtration (i.e., the host only allows certain phyllosphere microbial groups to colonize or persist), environmental filtration and various antagonistic or synergistic interactions between different phyllosphere microorganisms (e.g., competition, promotion, predation, etc.) [26,31]. According to whether environmental conditions change or not, the selection process can be divided into homogeneous selection and heterogeneous selection [26]. According to the degree of dispersal, the dispersal process can be divided into homogenizing dispersal and dispersal limitation [26]. Zhou and Ning constructed an iCAMP framework and developed it into an R software package to quantify the relative effects of homogeneous selection, heterogeneous selection, homogenizing dispersal and dispersal limitation, and ecological drift [26].

Up to now, only a few studies have explored the relative effects of different ecological processes (such as homogeneous selection, heterogeneous selection, homogenizing dispersal and dispersal limitation, etc.) in the assembly of the phyllosphere microbial community, and the effects of external disturbances on each ecological process of the assembly of the phyllosphere microbial community [30,32]. Previous studies have made some important scientific findings. For example, the relative influence of various ecological processes in the assembly of the phyllosphere microbial community will change with the growth and development of the host plant [33,34,35].

However, there are still two controversies present in previous studies. First, the main ecological process of the assembly of the phyllosphere microbial community is still debated. For example, random processes dominate the assembly of phyllospheric bacterial communities in rice [36]. In contrast, the determination process dominates the assembly of phyllospheric bacterial communities in 11 tree species, and the relative influence of homogeneous selection is greater than that of heterogeneous selection [37]. In addition, the assembly mechanism of the *Brassica napus* phyllosphere bacterial community and that of the *sorghum* phyllosphere fungi community change with its growth stage [34,35]. Second, whether and how external disturbances alter the relative effects of various ecological processes on the assembly of phyllospheric microbial communities is also still debated. For example, drought reduces the relative effects of dispersal limitation and homogeneous selection on the assembly of the rice phyllospheric bacterial community, but increases the relative effects of heterogeneous selection and ecological drift on the assembly of the rice phyllospheric bacterial community [36]. In contrast, drought has no significant effect on ecological processes such as dispersal limitation and homogenous selection of assembly of phyllospheric bacterial communities in alpine meadows [38]. These controversies relate to the fact that the various ecological processes of the assembly of the phyllosphere microbial communities change with seasonal changes/sampling times, plant species and varieties, etc. [25,35,37]. Therefore, it is necessary to strengthen the research on the assembly mechanism of the phyllosphere microbial community.

## 4. Factors Affecting the Structure of the Phyllosphere Microbial Community

The characteristics of plants and the ecological environment they live in will affect the growth, metabolism and immunity of plant leaves, etc., which can jointly affect the microbial community structure of the plant phyllosphere [9,39,40,41]. These influencing factors can be divided into abiotic factors (such as climatic conditions, geographical location and physical and chemical properties of soil, etc.) [12,42] and biological factors (such as phyllosphere morphological structure, physiological and biochemical characteristics, plant species and varieties, etc.) [37,43,44].

Climatic conditions mainly affect the structure of the phyllosphere microbial community in the following ways: (1) Climatic conditions may change the relative influence of various ecological processes on the assembly of the phyllosphere microbial community [36]. (2) Climate variables such as temperature and precipitation can affect the physicochemical properties of leaves [45,46] and soil [47,48], and thus affect the structure of the phyllosphere microbial community. Precipitation is also an important reservoir of phyllospheric microorganisms [49]. (3) Dust may be an important reservoir of the phyllosphere microbial community [50], and climatic variables such as precipitation and wind speed can affect dust. (4) Climate change in the historical period reshaped the spatial distribution pattern of vegetation. For example, the cooling of the last ice age caused forests to retreat to the south, while grasslands and deserts expanded from north to south and replaced the original position of forests. When the Holocene Great Warm period came, the warm and wet climate brought changes to the spatial distribution of vegetation in quite the opposite direction of the last ice age.

Geographical location (longitude, latitude and altitude) mainly affects the structure of a phyllospheric microbial community through the following ways: (1) Climate factors (mean annual temperature and precipitation, etc.), climate change (warming and precipitation changes, etc.), soil factors (soil organic carbon, etc.) and phyllosphere functional traits (phyllosphere area, etc.) generally change with geographical location [40,51]. (2) The spatial distribution pattern of contemporary natural vegetation is related to geographical location. For example, the Northern Tibet Plateau is distributed with alpine desert steppe, alpine steppe and alpine meadow from west to east [52]. (3) Changes in elevation (such as the overall uplift of the Tibetan Plateau, etc.) during historical periods reshape the spatial distribution of climate variables and vegetation [40].

Temporal variation (interannual variation and seasonal variation) mainly affects the structure of the phyllosphere microbial community in the following ways: (1) All ecological processes of the assembly of the phyllosphere microbial community change with time [33,34,35]. (2) Climatic conditions, soil physical and chemical properties, and phyllosphere morphology, structure, physical and chemical properties have significant seasonal and interannual variations [53,54].

Soil physical and chemical properties affect the phyllosphere microbial community structure mainly in the following ways: (1) Soil physical and chemical properties affect plant growth and development (seed germination, phyllosphere nitrogen and phosphorus content, photosynthesis, etc.) [55]. (2) The soil microbial community may also be an important repository of the phyllosphere microbial community [56,57].

Phyllosphere morphology, structure and physical and chemical properties mainly affect the community structure of phyllosphere microorganisms in the following ways: (1) The phyllosphere coat affects the contact area of phyllosphere microorganisms and plants and their habitat conditions, thus affecting the community structure of phyllosphere microorganisms [43]. (2) The thickness of the phyllosphere wax layer also affects the epiphytic ability of phyllosphere microorganisms. The thicker the phyllosphere wax layer, the less phyllosphere epiphytic microorganisms; conversely, the thinner the phyllosphere wax layer, the more phyllosphere epiphytic microorganisms [43]. (3) The phyllosphere cuticle has multiple functions for plant growth, such as reducing the loss of water and solute, reducing the temperature fluctuation of leaves by reflecting light, and preventing the invasion of pathogens, among which water repellency is particularly important in preventing the migration of microorganisms to the phyllosphere surface. At the same time, the function of the phyllosphere cuticle in limiting phyllosphere nutrient loss also has a great impact on the growth of the phyllospheric epiphytic microbial population [58]. (4) The moisture content of leaves affects the wetness of leaves and the dissolution and dispersal of nutrients inside leaves, thus affecting the fixed value and survival of foliar microorganisms in leaves [24,59,60]. (5) Most plants have many folds on the phyllosphere surface, and the low-lying areas of these folds affect water drop dispersal, thus providing better water conditions for the phyllosphere microorganisms. This phenomenon is consistent with the fact that the bacteria on the phyllosphere surface usually do not exist in the form of single cells, but tend to “live” together, and prefer to gather in the fold depression on the phyllosphere surface [61]. (6) Transpiration of leaves increases the relative humidity near stomata, which provides better water conditions for surrounding microorganisms [37]. (7) Leaves can secrete a small amount of nutrients such as glucose, fructose and sucrose, which provide a relatively sufficient carbon source for the phyllospheric microorganisms [62].

Plant species/varieties affect the structure of the phyllosphere microbial community mainly in the following ways: (1) The assembly mechanism of the phyllosphere microbial community [25], ecological strategies [44], morphological structure and physicochemical properties of leaves [57] are different among different plant species/varieties. (2) Parental “vertical transmission” is an important source of phyllosphere microorganisms [63], and the parental “vertical transmission” ability of different plant species/varieties may be different. (3) For the phyllosphere microbial communities of trees or perennial species, the number of migrations from soil microorganisms to phyllosphere microorganisms may be smaller due to distance and longer time for host adaptation [57,64].

The interactions between plant and phyllosphere microbes, or among phyllosphere microbes, can also regulate the community structure of phyllosphere microbes [31,65]. For example, extracellular polymers/surfactants secreted by foliar microorganisms can improve the water permeability of the phyllosphere cuticle, thereby increasing the interfoliar water content and the availability of dissolved compounds [66]. Siderophore and indoleacetic acid produced by phyllosphere microorganisms are important properties of these microorganisms [12,67]. They not only facilitate their colonization in the phyllosphere, but also give them effective biological control [12,67]. Iron acquisition is both a known virulence factor and a potential pathogen biological control feature, as non-pathogenic bacteria that produce iron carriers reduce iron availability and limit pathogen growth [12]. In addition, the phyllosphere microbial community structure is also affected by the rhizosphere microbial community structure and soil microbial community structure [12,68].

However, there are still two controversies which have arisen in previous studies. (1) The dominant factors of phyllosphere microbial community structure are still controversial. For example, air temperature is a major driver of the community structure of bacteria and fungi in the phyllosphere of wild strawberry [69]. Phyllosphere chemical properties (phyllosphere catalase and phosphorus content, etc.) dominate the community structure of rice phyllosphere epiphytic and endophytic bacteria [16]. Plant resistance to pathogens and phyllosphere chemistry (phyllosphere nitrogen and phosphorus content) jointly dominate the bacterial community structure of maize phyllosphere [10]. Phyllosphere iron, phyllosphere manganese, chlorophyll b and soil organic carbon jointly dominate the phyllosphere microbial community structure of rice [67]. In addition, temporal variation (seasonal variation and sampling month) dominate the community structure of *acacia* epiphytic bacteria [50]. (2) The relationship between phyllosphere microbial community structure and individual influencing factors is still controversial. For example, α diversity of phyllosphere epiphytes and endophytic bacteria in rice increased with the increase of phyllosphere nitrogen content [16], while α diversity of phyllosphere bacteria in maize decreased with the increase of phyllosphere nitrogen content [10]. The α diversity of phyllosphere epiphytes and endophytic bacteria increased with the increase of phyllosphere soluble sugar content in rice [16], while α diversity of phyllosphere bacterial is not related to phyllosphere soluble sugar content in maize [10]. These two controversies are related to the differences in geographical location and plant species in previous studies [60,70,71].

## 5. Effects of Climate Warming on Phyllospheric Microbial Community Structure

Climate warming is not only an important aspect of global change [45,47,72,73,74,75,76], but also an important external interference factor in the structure of the phyllosphere microbial community [38,77]. The phyllosphere microbial community not only regulates plant changes under short-term (years to decades) warming scenarios, but also long-term (centuries to millennia) warming scenarios [78]. Up to now, experimental studies on the effect of climate warming have mainly focused on plant rhizosphere and soil microbial community structure, while the influence of climate warming on plant phyllosphere microbial community structure and its driving mechanism have been largely ignored [77,79].

Up to now, based on the temperature gradient formed by geographical location (altitude gradient, etc.) or seasonal changes [1,40,50,80,81] and warming experiments [82,83,84], some scientific research results with theoretical value and practical guiding significance have been achieved on the impact of climate warming on the structure of the phyllosphere microbial community. For example, the phyllosphere microbial community structure of different dimensions (species and phylogeny, etc.) have different responses to climate warming [85]. Species and phylogenetic β diversity is often used to quantify the relative effects of different ecological processes (homogenous selection, heterogenous selection, homogenous dispersal, and dispersal limitation, etc.) on the assembly of the phyllosphere microbial community [34,36,37]. These ecological processes play important roles in revealing the maintenance mechanism of phyllosphere microbial diversity and the response mechanism to external disturbances. Second, the community structures of phyllosphere fungi and bacteria respond differently to climate warming [81,86]. This phenomenon may be related to their different community assembly mechanisms and nutritional strategies [33,40,69]. Phyllosphere fungi have a unique evolutionary life history (such as mycelium formation and survival, persistent spores, etc.), which enables them to enter directly into plant leaves by mechanical osmosis or secretory enzyme degradation of the cuticle and cell wall [40,87]. On the contrary, due to the absence of mycelium, the epiphytic bacteria cannot penetrate the cuticle of the leaf and enter the interior of the leaf like the phyllophyte fungi, but can only enter the interior of the leaf through the natural openings on the leaf surface and the mechanical damage [40,87]. Third, the community structure of epiphytic and endophytic microorganisms is different in response to climate warming, which may be related to their different living environmental conditions and assembly mechanisms [9,33,40,84]. At the macro and micro scales, there is significant environmental heterogeneity between the epiphytic and endophytic microbial communities (e.g., climatic conditions at the macro scale and biological/abiotic gradients at the micro scale) [40]. Compared with the surface of the leaves, there are better water conditions and a more abundant and stable nutrient supply inside the leaves [88]. The species pool and α diversity of epiphytic microbial communities tend to be larger than that of endophytic microbial communities [16,40,50]. These previous research results can provide a lot of theoretical basis with important scientific value for the response of phyllosphere microbial community structures to climate warming and their feedback to the ecosystem. However, because these findings are based on a limited number of plant species and genotypes (in fact, fewer than 30 published research papers have been published so far), caution is needed when extrapolating these findings [77,79].

The effect of climate warming on the structure of the phyllosphere microbial community is still controversial [79,82,89]. For example, there is no consensus on whether and how climate warming affects the α diversity of the phyllosphere microbial community. Studies have demonstrated a decrease [53,90] or no significant change [80,84,85,86]. Second, there is no consistent conclusion on whether climate warming changes the composition of the phyllosphere microbial community. Studies have demonstrated significant changes [53,84,89,90] or no significant effects [80,85,91]. Third, the impact of climate warming on potentially beneficial or pathogenic bacteria in the phyllosphere is also controversial. Some studies have found that climate warming reduces the relative abundance of phyllosphere microorganisms (such as *Sphingomonas*, *Methylobacterium* and *Rhizobium*) that are beneficial to plant growth, but increases the relative abundance of potential pathogenic bacteria (such as *Enterobacter*, *Erwinia*, *Acinetobacter*, *Buchnera*, *Wolbachia*, etc.) [89,92]. These indicate that a warming climate is likely to increase the risk of potential spread of plant or human pathogens in grassland/cropland ecosystems. Other studies have found that climate warming inhibits the growth and spread of potential pathogens in the phyllosphere [53]. Climate warming may also lead to the transfer of potential pathogens from the phyllosphere to higher altitudes [1]. These inconsistent or even completely opposite scientific findings are mainly related to the differences in plant species [77], initial phyllospheric microbial diversity [84], climatic conditions (mean annual temperature, etc.) [53], sampling time [53,83], and warming amplitude [83,93,94]. Therefore, how climate warming affects the phyllosphere microbial community structure and its driving mechanism have not been fully resolved, and further relevant studies are needed.

## 6. Conclusions

Some relevant studies have been carried out on plant phyllosphere microbial community structure, and a series of scientific research results have been obtained; for example, on the selection process and dispersal process, which jointly regulate the assembly of the phyllosphere microbial community. The phyllosphere epiphytic and endophytic microbial community structures have shown different responses to climate warming. The phyllosphere bacterial and fungal community structures also show different responses to climate warming. These results provide an important theoretical basis and scientific and technological support for the development of microbial ecology, farmland ecosystem management and coping with climate warming. However, previous studies have been inconsistent on the assembly mechanism of the phyllosphere microbial community (i.e., the relative effects of homogenous selection, heterogeneous selection, homogenous dispersal and dispersal limitation, etc.), the dominant driving factors of the change of the phyllosphere microbial community structure and the effects of climate warming on the plant phyllosphere microbial community structure. These controversies are related to the differences in plant species, plant phenology, geographical location and climatic conditions studied in the past. Therefore, there is a need to strengthen scientific research around these controversies.

## Data Availability

Not applicable.
